# Variation of Oxygenation Conditions on a Hydrocarbonoclastic Microbial Community Reveals *Alcanivorax* and *Cycloclasticus* Ecotypes

**DOI:** 10.3389/fmicb.2017.01549

**Published:** 2017-08-16

**Authors:** Fanny Terrisse, Cristiana Cravo-Laureau, Cyril Noël, Christine Cagnon, Alex J. Dumbrell, Terry J. McGenity, Robert Duran

**Affiliations:** ^1^IPREM UMR CNRS 5254, Equipe Environnement et Microbiologie, MELODY Group, Université de Pau et des Pays de l’Adour Pau, France; ^2^School of Biological Sciences, University of Essex Colchester, United Kingdom

**Keywords:** microbial ecology, active microbial communities, micro-diversity, oligotyping, high throughput sequencing, oil spill, hydrocarbon degradation, experimental ecology

## Abstract

Deciphering the ecology of marine obligate hydrocarbonoclastic bacteria (MOHCB) is of crucial importance for understanding their success in occupying distinct niches in hydrocarbon-contaminated marine environments after oil spills. In marine coastal sediments, MOHCB are particularly subjected to extreme fluctuating conditions due to redox oscillations several times a day as a result of mechanical (tide, waves and currents) and biological (bioturbation) reworking of the sediment. The adaptation of MOHCB to the redox oscillations was investigated by an experimental ecology approach, subjecting a hydrocarbon-degrading microbial community to contrasting oxygenation regimes including permanent anoxic conditions, anoxic/oxic oscillations and permanent oxic conditions. The most ubiquitous MOHCB, *Alcanivorax* and *Cycloclasticus*, showed different behaviors, especially under anoxic/oxic oscillation conditions, which were more favorable for *Alcanivorax* than for *Cycloclasticus*. The micro-diversity of 16S rRNA gene transcripts from these genera revealed specific ecotypes for different oxygenation conditions and their dynamics. It is likely that such ecotypes allow the colonization of distinct ecological niches that may explain the success of *Alcanivorax* and *Cycloclasticus* in hydrocarbon-contaminated coastal sediments during oil-spills.

## Introduction

Blooms of the marine obligate hydrocarbonoclastic bacteria (MOHCB) are usually observed after oil spills (Maruyama et al., 2003; Yakimov et al., 2007; Teramoto et al., 2013; Acosta-González et al., 2015; Duran and Cravo-Laureau, 2016; Yang et al., 2016a). Several studies have demonstrated that MOHCB are the main actors in the degradation of hydrocarbons in acute oil-pollution events (Bordenave et al., 2004b, Harayama et al., 2004; Yakimov et al., 2007; Brito et al., 2009; Said et al., 2010; Coulon et al., 2012; Gutierrez et al., 2013; Sanni et al., 2015), thus occupying a specific trophic niche in the carbon cycle. Among them, members of the genera *Alcanivorax* and *Cycloclasticus* are almost always detected in samples from marine environments around the world after oil input (Harayama et al., 2004; Yakimov et al., 2007; Brito et al., 2009; Said et al., 2010; Gutierrez et al., 2013; Louvado et al., 2015; Jeanbille et al., 2016; Kleindienst et al., 2016). Members of these ubiquitous genera occupy distinct trophic niches, where usually the aliphatic hydrocarbon degrader *Alcanivorax* blooms first, followed by the (poly-)aromatic hydrocarbon degrader *Cycloclasticus*, as polyaromatic hydrocarbons (PAH) are less amenable to degradation (Head et al., 2006).

Despite our improved knowledge of the ecology of MOHCB, their behavior when confronted with fluctuations in environmental parameters is far from understood. Coastal marine sediments are subjected to fluctuating oxygenation and thus redox conditions from tidal cycles, diurnal cycles (and thus photosynthetic oxygenation), and macrofaunal burrowing activities that in turn affect microbial degradation processes (McKew et al., 2013; Cravo-Laureau and Duran, 2014; Duran et al., 2015a,b). The time span of these oxygen intrusions often varies from few minutes to several hours (Wakeham and Canuel, 2006; Militon et al., 2015, 2016). Previous studies demonstrated that anoxic/oxic oscillations promote organic matter biodegradation (Abril et al., 2010), and more particularly hydrocarbon degradation (Cravo-Laureau et al., 2011; Vitte et al., 2011, 2013). However, the microbial processes underlying the biodegradation of hydrocarbons within the anoxic/oxic transitional zone is not well understood (Cravo-Laureau et al., 2011; Cravo-Laureau and Duran, 2014), especially for MOHCB subjected to such extreme fluctuating conditions.

The interaction of microorganisms with their environment is a key question in microbial ecology. Microorganisms have developed several metabolic and behavioral strategies to survive under different environmental conditions (Shade et al., 2012), which include physiological versatility and plasticity (Dolla et al., 2006; Fourcans et al., 2008; Evans and Hofmann, 2012) and dormancy (Lennon and Jones, 2011). Alternatively, the adaptation could involve ecotypes, subpopulations with specialized adaptations to microenvironments (Hunt et al., 2008; Coleman and Chisholm, 2010). The ecotype formation is an adaptive process allowing subpopulations to occupy an ecological niche, a first step in the speciation process (Wiedenbeck and Cohan, 2011). An impressive example is provided by the multiple ecotypes of the cyanobacterium *Prochlorococcus*, which was found in different abundances according to environmental factors such as light, temperature, and phosphate and nitrate contents (Martiny et al., 2009). Such ecotype diversification supports the success of *Prochlorococcus* as a dominant phototroph in oligotrophic seawater (Martiny et al., 2006, 2009). Ecotypes are recognized as phylogenetic clusters occupying a specific habitat. The existence of ecotypes has be shown by the habitat specificity of 16S rRNA sequences, as demonstrated for *Synechococcus* adapted to different temperatures (Melendrez et al., 2011) and for the PAH-degrading *Alteromonas* adapted to different depth of seawater (Math et al., 2012). Based on the oligotyping approach, a computational method that reveals the micro-diversity within sequences clustering in a single OTU (Eren et al., 2013), Kleindienst et al. (2016) proposed ecotypes for the hydrocarbon-degraders *Cycloclasticus, Colwellia* and *Oceanospirillaceae* adapted to hydrocarbon gradients.

In order to test the hypothesis that the success of MOHCB, particularly the most ubiquitous *Alcanivorax* and *Cycloclasticus*, to colonize distinct niches is based on ecotype diversification, an experimental ecology approach that offers the possibility to test ecological hypotheses under controlled conditions (Cravo-Laureau and Duran, 2014) was implemented. A microbial hydrocarbon-degrading community was maintained in bioreactors and exposed to different oxygenation regimes including permanent anoxic, anoxic/oxic oscillation and permanent oxic conditions. The bacterial diversity dynamic was assessed by16S rRNA gene transcript sequences (using high throughput sequencing technology) and the micro-diversity of *Alcanivorax-* and *Cycloclasticus-*related sequences was examined. First, oligotypes were defined and correlated with the redox conditions, suggesting the presence of subpopulations. Then, single nucleotide difference analysis confirmed the presence of cohesive subpopulations for the different oxygenation conditions representing specific ecotypes. The distribution of the ecotypes throughout the incubations explained the dynamics of *Alcanivorax* and *Cycloclasticus* during the anoxic/oxic oscillations.

## Materials and Methods

### Hydrocarbon-Degrading Microbial Community

Intertidal sediments were collected at the tidal basin Aber-Benoît (Tréglonou, France, 48° 33′12.40″N; 4° 32′8.69″W) in January 2012 as previously described (Stauffert et al., 2013; Cravo-Laureau et al., 2015). A hydrocarbon-degrading microbial community was obtained by exposing sediments to oil (24 ± 4 mg/g of crude oil) during 250 days in mesocosms with simulated tidal cycles as previously described (Stauffert et al., 2014a, 2015). When biodegradation was observed (80% of TPH removal, *n*C_17_/phytane index = 0.3) by quantification of the hydrocarbon content (Duran et al., 2015a), 2400 cm^3^ of surface sediments (6 cm thick) were collected in order to prepare a slurry for the inoculation of bioreactors. Sediments were maintained in natural seawater in the dark at 4°C, bubbling surface water with filtered air (PTFE filters of 0.2 μm pores size, Fisher Scientific) for 10 days before slurry preparation. The slurry was obtained by homogenization of 20% (w/v) sediments with natural filtered sea water (MFTM filter of 0.45 μm pores size, Millipore), containing 3.7 mM NH_4_Cl, 24 mM NaHCO_3_, 3.2 mM potassium phosphate buffer (Sambrook and Russell, 2001) and 1 mL/L V7 vitamin (Pfennig et al., 1981), at pH 7.2.

### Bioreactors System and Experimental Set Up

The slurry (1.8 L) was distributed in nine bioreactors (with a working volume of 2 l) to apply the three following conditions in triplicate: anoxic/oxic oscillation, permanent oxic and permanent anoxic conditions as detailed (Terrisse et al., 2015). The incubations were carried out for 15 days in batch conditions, with stirring (250 rpm, Stuart SS20), in the dark and at room temperature (ranging from 17 to 24°C, InPro6800 sensors, Mettler Toledo International Inc.). Bioreactors were maintained for 5 days, under oxic or anoxic conditions (for the permanent anoxic and the anoxic/oxic oscillation conditions) for a microbial community stabilization period, before addition of crude oil and starting anoxic/oxic oscillations for the oscillating conditions. At day 5, Russian export blend crude oil (REBCO) was added with a concentration of 21.2 ± 5.7 mg/g of dry weight sediments. The REBCO is an Ural type crude oil, distilled at 110°C to eliminate the more volatile hydrocarbon compounds. This oil contained 59.9% of saturated and 24.8% of aromatic hydrocarbons, 10.2% of resins and 5.1% of asphaltenes. In order to be close as possible to conditions prevailing in the environment, where oxygen pulses often occur at timescales of minutes to hours (Wakeham and Canuel, 2006; Militon et al., 2015, 2016), anoxic/oxic oscillations consisted of an alternation of anoxic periods with two 1-day periods of aeration performed at days 7 and 10. Aeration periods were produced by injection of filtered air (Acro^®^37 TF Vent Device with a 0.2 μm PTFE Membrane, Life Sciences) using air pumps (flow rate of 70 L/h, Rena air 50) into the gas and aqueous phases, generating bubbling in the latter. Permanent oxic conditions were produced in the same way. Periods of anoxic conditions were achieved by stopping aeration, sealing, closing the system and creating a slight overpressure with nitrogen gas. The same process was used to achieve the permanent anoxic conditions. Dissolved oxygen, temperature (InPro6800 sensors, Mettler Toledo International Inc.), pH and redox potential (InPro 4260i/SG/225 sensors, Mettler Toledo International Inc.) were measured twice a day.

### Sample Collection

Samples were collected with a sterile syringe (TERUMO Corporation), connected to bioreactors by a Norprene tube, for chemical and biological analyses. The sampling system was purged before each sampling. 10 mL samples were collected for chemical analysis from each reactor along the incubation at 5.4 (i.e., 10 h after oil addition), 7, 8, 10, 11, and 15 days of incubation. Sampling was performed before switching condition. Samples were stored in amber glass bottles with polytetrafluoroethylene stoppers (WHEATON) at -20°C. For molecular analyses 1.5 mL of slurry was collected and immediately mixed with 190 μL of RNA stabilization solution (the RNA stabilization buffer was prepared as follow: 5 mL of phenol were mixed with 5 mL of 1 M Na-acetate buffer pH 5.5, then the two phases were separated by centrifugation at 4000 × *g* for 3 min. The phenolic phase was finally added to 95 mL of pure ethanol) to preserve rRNA transcripts integrity. Samples with RNA stabilization solution were homogenized and centrifuged at 10000 × *g* for 5 min at 4°C (Jouan MR 1812). The supernatant was then removed and tubes containing pellets were introduced immediately in liquid nitrogen and stored at -80°C. Samples were collected in triplicate from each reactor at days 0, 5 (before oil addition), 5.4 (10 h after oil addition), 7, 8, 10, 11, and 15.

### Hydrocarbon Analysis

Samples were filtered through glass microfiber filters (grade GF/F, 0.7 μm pore size, Whatman) separating sediments and aqueous phases. Sediments analyses were performed according to Stauffert et al. (2013) including perdeuterated eicosane (*n*-C_20_d_42_) and five perdeuterated PAHs (d_8_-naphtalene, d_10_-biphenyl, d_10_-phenanthrene, d_12_-chrysene and d_12_-benzo(*a*)pyrene; Sigma Aldrich, United States) as internal standards. Hydrocarbons were extracted with dichloromethane, dried over Na_2_SO_4_ and concentrated to 2 mL as previously described (Stauffert et al., 2015). Hydrocarbons were analyzed by gas chromatography coupled with mass spectrometry (GC-MS) as previously described (Stauffert et al., 2013).

Aqueous samples were analyzed after addition of internal standards (perdeuterated PAHs), extracted according to the Stir Bar Sorptive Extraction protocol and analyzed by SBSE-GC/MS as previously described (Stauffert et al., 2013).

Chemstation software was used for determining concentrations of hydrocarbons. The total petroleum hydrocarbons and the target molecules of *n*-alkanes and PAHs were quantified relatively to the perdeuterated eicosane and PAHs (internal standards) using calibration curves of REBCO crude oil (from 0.05 to 5 mg/mL), of *n*-alkanes (TRPH Standard from 0.5 to 50 μg/mL, Ultra Scientific, Florida) and of PAHs (CUS-9306 from 0.25 to 25 μg/mL, LGS Standards, France), respectively.

### Total DNA and RNA Co-extraction

Genomic DNA and RNA transcripts were co-extracted using the commercial RNA PowerSoil^®^Total RNA Isolation Kit (MoBio Laboratories). Extractions were performed according to the manufacturer’s instructions with a slight modification: step 4 was amended by suspending nucleic acids in 100 μL of SR5 buffer (supplied in the kit) after precipitation with ethanol 70% v/v. The separation and purification of DNA and RNA were performed with the commercial Allprep DNA/RNA Mini Kit (QIAGEN) following the manufacturer’s recommendations. RNA and DNA were separately eluted in 100 μL of free DNase/RNase sterilized MilliQ water. RNA extracts were treated with Turbo DNA free kit (Ambion, Applied Biosystems) according to the manufacturer’s instructions to ensure total elimination of DNA. DNA total elimination from RNA extracts was checked by negative 16S rDNA PCR amplification on the extracts. The quality and the size of DNA and RNA obtained were verified by electrophoresis on a 1% w/v agarose gel in Tris-Borate-EDTA buffer. RNA quality and concentration were also investigated by micro-capillary electrophoresis using RNA 6000 Nano LabChips kit and an Agilent 2100 Bioanalyzer (Agilent Technologies). RNA Integrity Numbers (RIN) between 7 and 8.5 were obtained for the RNA extracts, indicating good quality. The DNA and RNA extracts were aliquoted and stored at -80°C.

### Reverse Transcription of RNA

The synthesis of a complementary DNA strand (cDNA) from a RNA template strand was performed by reverse transcriptase as previously described (Stauffert et al., 2014b). Equipment and solutions used were certified RNase and DNase free or previously treated with DEPC (diethylpyrocarbonate). The reaction (final volume of 20 μL) was carried out with 10–60 ng of RNA per sample, adding 40 U of RNase OUT (Invitrogen by Life Technology), 0.5 mM of dNTPs, 10 ng/μL of random hexamers (Roche), 0.01 M DTT, 1 × enzyme buffer and 200 U of the Moloney murine leukemia virus reverse transcriptase (M-MLV, Invitrogen by Life Technology), following suppliers’ advice. The cDNA products were then used directly in a PCR reaction or stored at -80°C.

### Quantitative-PCR of 16SrDNA on DNA and cDNA

DNA and cDNA were amplified using primers 338F (5′-CTCCTAC GGGAGGCAGCAGT-5′) and 518R (5′-GTATTACCGCGGCTGCTG-3′) targeting bacterial 16S rRNA as previously described (Giloteaux et al., 2010). LightCycler^®^480 SYBR GREEN I Master was used to prepare 10 μL reactions containing 5 μL of 2 × MasterMix, 0.4 μM of each primer, 2.5 μL of 1/100 diluted DNA or cDNA extracts. Quantitative Polymerase Chain Reaction (Q-PCR) was performed with the Roche LightCycler 480 Real Time PCR system. The cycling program was as follows: 95°C for 5 min followed by 40 cycles with denaturation step at 95°C for 15 s, hybridization step at 60°C for 15 s and elongation step at 72°C for 20 s. At the end of the program, an increase of the temperature from 64°C to 97°C provided the melting curves, informing on the amplification quality. The LightCycler 480 Software was used to analyze fluorescence. 16S rRNA copy numbers per μL from DNA or cDNA amplifications were quantified relatively to calibration curves as previously described (Paissé et al., 2012).

### Sequencing and Data Analysis

High-throughput sequencing analysis, targeting bacterial 16S rRNA, was performed from 0.1 μg cDNA at days 0 (mix from 3 extract samples for day 0), 5, 5.4, 7 (under oxic and anoxic conditions), and 8, 10, 11, 15. Amplicon 454-pyrosequencing was performed by the Molecular Research DNA laboratory (MR DNA, Shallowater, TX, United States) following process originally described by Dowd et al. (2008). The 16S universal bacterial primers 27Fmod (5′-AGRGTTTGATCMTGGCTCAG-3′) and 519Rmodbio (5′-GTNTTACNGCGGCKGCTG-3′) were used, targeting V1-V3 variable regions. A single-step PCR using HotStarTaq Plus Master Mix Kit (Qiagen, Valencia, CA, United States) was used with the following conditions: 94°C for 3 min, followed by 28 cycles of 94°C for 30 s, 53°C for 40 s and 72°C for 1 min, after which a final elongation step at 72°C for 5 min was performed. The different PCR products were mixed in equal concentrations before purification using Agencourt Ampure beads (Agencourt Bioscience Corporation, Beverly, MA, United States). Roche 454 FLX titanium instruments and reagents were used to perform the sequencing following manufacturer’s guidelines.

The open source software QIIME (Quantitative Insights Into Microbial Ecology) was used for sequence read analysis of the bacterial 16S rRNA gene sequence (Caporaso et al., 2010). From the raw sequencing output, data cleaning was initially performed (Quince et al., 2009) by denoising of the data and by eliminating chimera and sequences with length under 450 bp, with mistakes in primers sequences or with homopolymers. Sequences reads were aligned on a sequence length of 400 bp. The data OTU picking was carried out according to the Usearch method (Edgar, 2010) defining reference sequences at the similarity threshold of 0.97. The taxonomy assignment was performed comparing reference sequences to a reference database of known 16S rRNA genes, the Ribosomal Database Project (RDP) database. Results of the number of times an OTU was found in each sample were tabulated and the taxonomic predictions were added for each OTU. The OTU abundances for each sample were rarefied to the same number corresponding to the minimum read number observed for a sample (3,102 sequences). This normalization of the OTU abundance data per sample was performed with the package vegan (Oksanen et al., 2013; Version: 2.0–10) with R software. The complete dataset was deposited in the NCBI Sequence Read Archive (SRA) database and is available under the Bioproject ID PRJNA383383.

### Statistical Analyses

The effects of the incubation conditions (anoxic/oxic oscillation, permanent oxic or permanent anoxic conditions), time and interactions among these factors, on the variability of gene expression rates, biodegradation ratios, coverage values and univariate diversity indexes (richness and Shannon’s diversity estimated with R and Mothur software), were tested by two multifactorial analysis of variance. A linear mixed model was performed with the effects of the incubation condition and the time (and their interactions) as fixed factors and a random factor to take into account the effect of repeated measurements over time, corresponding to the non-independent nature of the various samples in bioreactors (lme function in R package nlme). A second linear model was conducted by testing the same factors as fixed factors but without taking into account the random factor (lm function in R). To test the effect of the random factor, analysis of variance (Kunin et al., 2008) has shown no significant difference between these two models, the linear model without repeated measurement was chosen. The logarithmic transformation of the numeric variable was sometimes necessary in order that the residuals follow a normal distribution. ANOVA and Tukey’s HSD tests (Honest Significant Difference) were carried out following the linear models to compare between each other the different modalities of the various factors and highlight significant differences. Permutational multivariate analysis of variance (PerMANOVA) tested significance among incubation conditions and time as fixed factors and the random factor to take into account the effect of repeated measurements over time, with 16S rRNA sequencing data (based on dissimilarity matrix of the Bray-Curtis distances). Bacterial community composition (16S rRNA sequencing data) under the different incubation conditions was analyzed using non-metric multidimensional scaling (nMDS) implemented in Primer 6 (version 6.1.16). For nMDS ordinations, the Bray-Curtis distance was used to generate dissimilarity matrices. Confidence ellipses were based on cluster analyses (Primer 6 software; version 6.1.16). Schematic representations of OTU distribution according to incubation conditions and time were drawn with ade4 package in R software (Dray and Dufour, 2007).

### *Alcanivorax* and *Cycloclasticus* Micro-Diversity Analyses

*Alcanivorax* and *Cycloclasticus* were perfect candidates for micro-diversity analysis, since they were largely present within our samples playing a key role in oil-degrading microbial assemblage. First, subpopulations were examined by oligotyping (Eren et al., 2013). Oligotyping analysis generates oligotypes by systematically identifying nucleotide positions that represent information-rich variation among closely related sequences. The identification of nucleotide positions of interest was performed using the Shannon entropy (Shannon, 1948). A total of 53,842 and 26,120 sequences were extracted respectively from the dominant OTUs (represented by more than 100 sequences) of *Alcanivorax* (ID.A0; ID.A37; ID.A56; ID.A5335; ID.A8753; ID.A9793) and *Cycloclasticus* (ID.C3; ID. C178; ID.C834; ID.C1181; ID.C1762; ID.C4039; ID.C5592; ID.C7005; ID.C7228; ID.C8234; ID.C8389; ID.C9138; ID.C9204; ID.C9738; ID.C9747). An alignment was performed against the SILVA database (release 128) and sequences were trimmed to a consistent start and end position. Using the Shannon entropy analysis, a total of 35 and six information-rich positions contributing to the *Alcanivorax* and *Cycloclasticus* oligotypes were identified. To reduce the noise, only oligotypes that occurred in more than 1% of the reads for at least one sample in which the most abundant unique sequence represented more than 0.01% of all reads, were retained. The analysis of the distribution of oligotypes was conducted from the raw counts data without normalization, because the identified oligotypes presented low diversity in each sample. Then, in order to resolve in depth the distribution of each genus within the different oxygenation conditions, a new clustering of *Alcanivorax* and *Cycloclasticus* was performed using the swarm algorithm (single-linkage clustering method) implemented in Qiime (Caporaso et al., 2010; version 1.9.1), with a local clustering threshold of 1 (*d* = 1). Thus, clusters of sequence were obtained differing by one nucleotide defining swarm OTUs that correspond to ecotypes. Only swarm OTUs composed of more than 100 sequences were retained. The distribution of these swarm OTUs was represented in heatmaps carried out with DECIPHER (R package; Wright, 2016) using the *log*-transformed data in order to deal with the skewed and wide distribution of the raw data. Since swarm OTUs represented cohesive subpopulations specifically correlated with an oxygenation condition they were defined as ecotypes.

## Results and Discussion

### Redox Status and Hydrocarbon Degradation

The redox and oxygen saturation, followed during the 15 days bioreactor-incubation of the hydrocarbon-degrading bacterial community, indicated that the hydrocarbon-degrading microbial community was effectively subjected to three oxygenation regimes, namely permanent oxic, permanent anoxic and anoxic/oxic oscillation conditions (Supplementary Figure 1). Under anoxic/oxic oscillation condition, the oxygenation periods at days 7 and 10 were characterized by a liquid phase with oxygen saturation above 50% and redox potentials higher 15 mV while the anoxic periods were characterized by a liquid phase with oxygen saturation at 0% and redox potentials bellow -200 mV as observed for the permanent anoxic condition. Such redox oscillations modify the availability of oxidants and reductants, which in turn influences microbial community structure (Lipson et al., 2015) and metabolic activities (DeAngelis et al., 2010), supporting increased microbial activity (Fenchel and Finlay, 2008). Previous studies demonstrated that anoxic/oxic oscillations promote organic matter biodegradation (Abril et al., 2010), and more particularly hydrocarbon degradation (Cravo-Laureau et al., 2011; Vitte et al., 2011, 2013). In our study the *n*-C_17_/pristane ratio (McKenna and Kallio, 1971) and phenanthrene/dimethylphenanthrene ratio (Michel and Hayes, 1999), used as indexes reporting the biodegradation for *n*-alkane and PAH respectively, indicated that the most efficient biodegradation for *n*-alkanes was under anoxic/oxic oscillation condition while similar efficiency was observed for PAH biodegradation under permanent oxic and anoxic/oxic oscillation conditions at the end of incubation (Supplementary Figure 2). It was notable that *n*-alkanes were progressively depleted under anoxic/oxic oscillation conditions while PAH biodegradation was more efficient during the aeration periods. This observation suggests co-stimulation of aerobic and anaerobic metabolism for the biodegradation of *n*-alkanes as proposed for organic matter degradation (Abril et al., 2010) while PAH biodegradation involved exclusively aerobic metabolisms. The latter implies that PAH-degrading microorganisms developed strategies for their maintenance under anoxic conditions. Such an assumption was supported in our study by a significant correlation (Pearson correlation index *r* = 0.904, *p*-value < 0.05) between PAH biodegradation index (phenanthrene/dimethylphenanthrene ratio) and expression rate (rRNA transcripts copies number/rRNA genes copies number) of 16S rRNA gene (Supplementary Figure 3) indicating growth stimulation of PAH-degrading microorganisms after oxygen input. It is thus likely that PAH-degrading microorganisms were in dormancy during the anoxic periods of the anoxic/oxic oscillation condition. Dormancy strategies play crucial role in microbial community stability in fluctuating environmental conditions, particularly by providing seed bank maintaining metabolic diversity through various mechanisms including persistent-subpopulation ecotypes, niche complementation and functional redundancy (Lennon and Jones, 2011; Shade et al., 2012). Dormancy strategies have been evidenced for marine microbial communities in coastal areas (Campbell et al., 2011; Hugoni et al., 2013). However, in a previous study we demonstrated that *rhd* gene transcripts, encoding ring hydroxylating dioxygenase involved in the first step of PAH biodegradation, were continuously produced during anoxic/oxic oscillation conditions including the anoxic periods (Vitte et al., 2013) suggesting a metabolic activity of some PAH-degrading populations even in absence of oxygen. It is likely that some of the hydrocarbon-degrading populations may adopt a dormancy strategy while another part would remain metabolically active. Determining the dynamic of the hydrocarbon-degrading populations during fluctuating redox conditions at a fine taxonomic scale may help to unveil the microbial processes involved in the adaptation to fluctuating redox conditions.

### Influence of Oxygenation Regimes on the Overall Bacterial Community Organization

**FIGURE 1:**
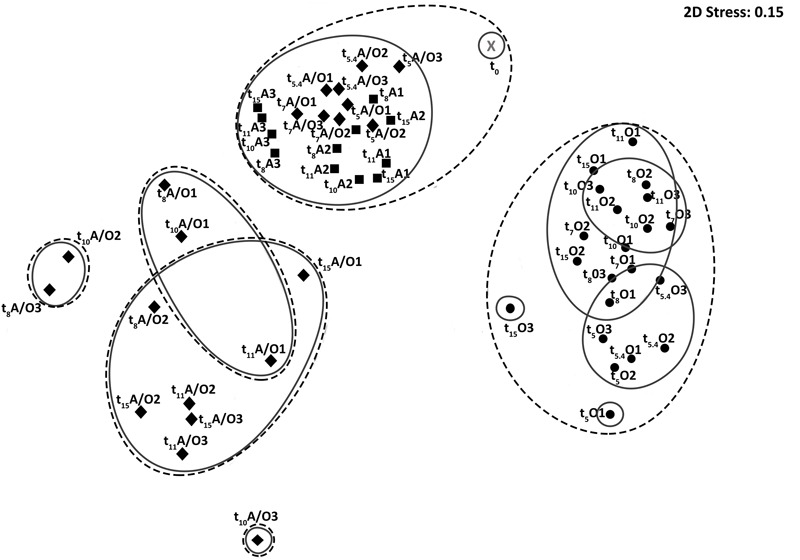
Comparison of the metabolically active bacterial community structures. Non-parametric Multidimensional Scaling (nMDS) based on pyrosequencing data from 16S rRNA gene transcripts obtained during bioreactor incubations under permanent anoxic condition (A, filled squares), anoxic/oxic oscillation condition (A/O, filled diamonds) and permanent oxic condition (O, filled circles). Solid and dotted black lines represent 30 and 20% of Bray-Curtis similarity respectively. Analyses were performed in biological triplicates, which are indicated by a number. Samples are named by time (t0, 5, 5.4, 7, 8, 10, 11, and 15 days), condition (A, anoxic; A/O, anoxic/oxic oscillation and O, oxic) and replicate number (1, 2, and 3).

**FIGURE 2:**
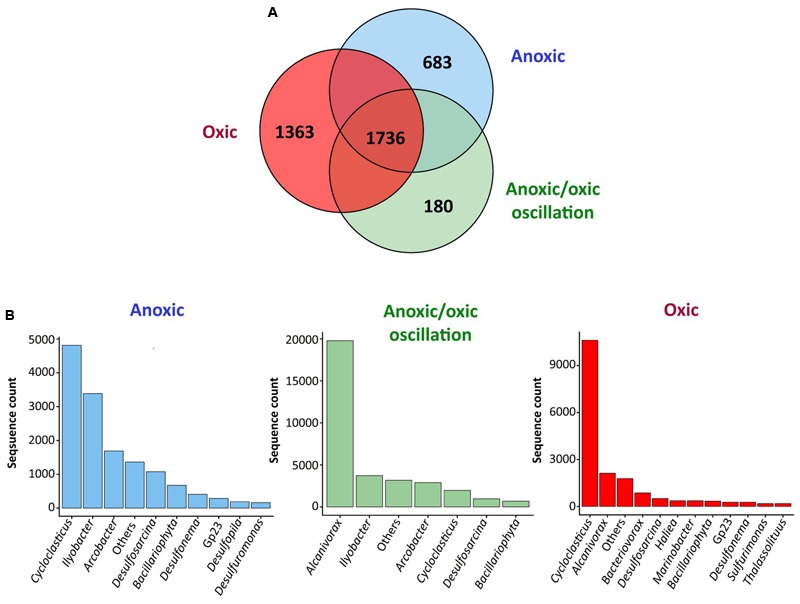
Comparison of bacterial communities from the different conditions. **(A)** Venn diagram showing the number of shared OTU_97_s between all conditions and the number of specific OTU_97_s for each condition. **(B)** Relative abundances of the specific OTU_97_s for each condition. The analysis was performed at the genus level applying a threshold similarity of 97% for OTU identification (OTU_97_s). The analysis includes all samples covering the whole incubation period. The analysis is based on biological triplicates. The other^∗^ group combine the genera related to rare OTUs that are represented by less than 1% of total sequences per sample. The relative abundances of the OTU_97_s belonging to the “other group” are presented in Supplementary Figure 4.

In order to determine the mechanisms underlying the behavior of hydrocarbon-degrading bacterial communities under different oxygenation regimes, bacterial community compositions were characterized at different incubation times for the three oxygenation conditions by high throughput sequencing of 16S rRNA gene transcripts, thus assessing active bacterial communities. After trimming and rarefication 3,102 sequences were obtained per sample corresponding between 177 and 600 OTU_97_s at the species level (97 % similarity threshold) per sample. The characteristics of the revealed diversity are presented in Supplementary Table 1. Good’s coverage were above 0.90 indicating that the number of sequences per sample was sufficient to describe the accessible diversity. The α-diversity indexes [Richness (*R*) and Shannon (*H*)] varied significantly with the condition (PerMANOVA, *p*-value < 0.001), the time (PerMANOVA, *p*-value < 0.005 and <0.05 respectively) and time variation was different between conditions (PerMANOVA, *p*-value < 0.001). Furthermore, *R* and *H* were higher for permanent anoxic and permanent oxic conditions (PerMANOVA, *R* > 400 OTUs; *p*-value < 0.001; *H* > 3.6 and 4.0 respectively) than for anoxic/oxic oscillating conditions after the first period of aeration (*R* < 250 OTUs; *H* < 2.5). These observations corroborated the effect of oxygen regime on bacterial community organization, which was further supporter by nMDS analysis showing three main clusters (i) anoxic/oxic oscillating communities, (ii) permanent oxic communities and (iii) anoxic/oxic oscillating + permanent anoxic communities (**Figure 1**). The Venn diagram comparing bacterial communities revealed that 1,736 OTU_97_s were shared between the three conditions, which represent 46, 49, and 56% of total OTU_97_s for the permanent anoxic, permanent oxic and anoxic/oxic oscillating conditions respectively (**Figure 2**), suggesting that a large proportion of microorganisms have the capacity to tolerate the different oxygenation regimes. It is notable that these OTU_97_s represented above 70% of the sequences retrieved in each condition corresponding thus to the most abundant OTU_97_s. Among these shared OTU_97_s, the most abundant were related to the MOHCB genera *Alcanivorax* (37%) and *Cycloclasticus* (28%), some species of which related species have been described as aerobic and microaerobic (Dyksterhouse et al., 1995; Yakimov et al., 1998; Lai et al., 2013). Regarding the specific OTU_97_s for each condition, low abundant OTU_97_s that may play an important role in the microbial assemblage organization and functioning were found. Among the less abundant OTU_97_s (Supplementary Figure 4), sequences related to genera know to present hydrocarbon degradation capacities were found, such as *Thalassolituus* (Yakimov et al., 2007), *Marinobacter* (Duran, 2010), *Marinobacterium* (Sherry et al., 2013), *Pseudomonas* (Paisse et al., 2011) and *Desulfobacterium* (Paissé et al., 2008). Interestingly, the specific sequences were also related to *Cycloclasticus* and *Alcanivorax* OTU_97_s, which dominated the less abundant OTU_97_s in each condition, except in the permanent anoxic condition where specific *Alcanivorax* OTU_97_s were observed at low abundance (<50 sequences). It is important to note that under permanent anoxic conditions the specific OTU_97_s represented 2% of the sequences corresponding thus to rare OTU_97_s (**Figure 2**), which were dominated by *Cycloclasticus* and *Ilyobacter.* Members related to *Ilyobacter* have been found specialized in the degradation of hydroaromatic compounds under anoxic conditions (Brune et al., 2002). Both *Alcanivorax* and *Cycloclasticus* are widely distributed, detected in various marine environments including surface water, hydrothermal vents, deep sea water bodies, coastal and mudflat sediments (Bordenave et al., 2004a, 2008; McKew et al., 2007; Yakimov et al., 2007; Staley, 2010; Paisse et al., 2011; Coulon et al., 2012). It is not surprising to observe these microorganisms together because they use different hydrocarbon substrates as carbon and energy sources (McKew et al., 2007; Coulon et al., 2012). More intriguing is their presence in anoxic conditions, particularly for *Cycloclasticus*, species of which related have been described as aerobic bacteria. However, *Cycloclasticus* species have been detected in bio-irrigated coastal marine sediments (Montgomery et al., 2008) and isolated from marine polychaete burrows in an intertidal mudflat (Chung and King, 2001), suggesting that related members of this genus have the capacities to withstand fluctuating environmental conditions. Additionally, *Cycloclasticus* relatives have been found active in methane-enriched microcosms (Sauter et al., 2012) and environments (Redmond and Valentine, 2012). Recently, the metabolic versatility of *Cycloclasticus* in the degradation of hydrocarbons was demonstrated (Rubin-Blum et al., 2017) and *Cycloclasticus* ecotypes, specific for the deep sea methane-rich hydrocarbon plume arose with the *Deepwater Horizon* oil spill, have been proposed (Kleindienst et al., 2016) based on the oligotyping approach (Eren et al., 2013).

### Influence of Oxygenation Regimes on *Alcanivorax* and *Cycloclasticus* Populations

**FIGURE 3:**
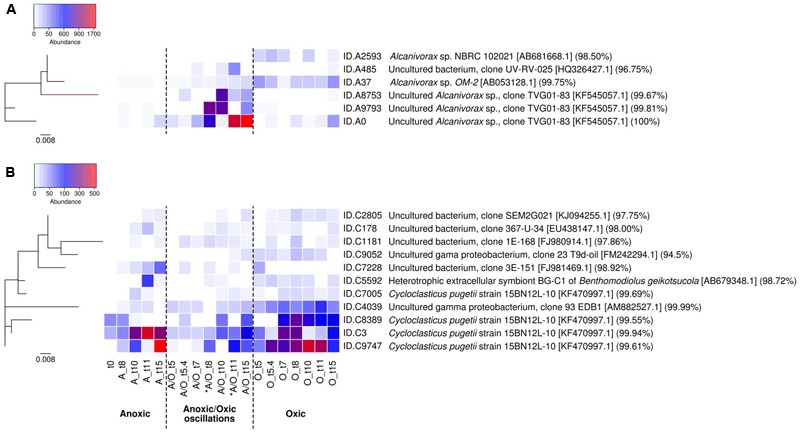
Relative abundances of *Alcanivorax* OTU_97_s **(A)** and *Cycloclasticus* OTU_97_s **(B)** according to oxygenation regimes. The heatmap was performed at the species level applying a threshold similarity at 97% for OTU identification (OTU_97_s). The OTU_97_s abundances were normalized by rarefication to the lowest read count of a sample. Phylogenetic trees are shown on the left (the bars represent 0.8% estimated sequence divergence). ID sequences and the highest BLAST hits are indicated on the right. Color legend shows OTU_97_s abundance. The analysis is based on biological triplicates. ^∗^ indicates oxygenation period under anoxic/oxic oscillation condition.

The distribution of *Alcanivorax* and *Cycloclasticus* OTU_97_s within the different oxygenation conditions was examined in order to determine whether specific OTU_97_s explain their survival under the distinct conditions. *Alcanivorax* and *Cycloclasticus* were represented by six and eleven OTU_97_s respectively, the relative abundance of which varied between the different conditions during the incubation period (**Figure 3**). It is important to note that the distribution of *Alcanivorax* and *Cycloclasticus* OTU_97_s did not show difference between biological replicates (PerMANOVA *p*-value > 0.05). *Alcanivorax* and *Cycloclasticus* genera adopted different strategies to face fluctuating conditions: *Alcanivorax* taking advantage of brief favorable condition (oxygenation) for growth under fluctuating conditions whereas *Cycloclasticus* requiring stable conditions to attain its maximun abundance (**Figure 3**).

*Alcanivorax* was the most abundant genus under anoxic/oxic conditions despite the fact that relatives were not detected in the metabolically active bacterial communities at the beginning of the incubations. They were below the detection limit under the permanent anoxic incubation. *Alcanivorax* related species have been described as nitrate reducing bacteria (Yakimov et al., 1998), but here the redox potential was obviously too low to allow their development. Two OTU_97_s (ID.A37 and ID.A2593) were found abundant under the permanent oxic condition and poorly represented under the other conditions (**Figure 3A**). These OTU_97_s were closely related to *Alcanivorax* sp. NBRC 102021 (acc. no. AB681668) isolated from seawater for ID.A2593 and *Alcanivorax* sp. OM-2 (acc. no. AB053128) isolated from oiled marine sediments for ID.A37. Interestingly, four *Alcanivorax* OTU_97_s (ID.A0, ID.A9793, ID.A8753 and ID.A485), although they were either not detected or in very low abundance under the permanent oxic condition, were found with high relative abundances following periods of aeration under the anoxic/oxic oscillating condition (**Figure 3A**). This observation suggested that an episodic presence of oxygen was favorable for *Alcanivorax* growth. Episodic intrusion of oxygen in anoxic zones has been shown to support aerobic metabolism in typical anoxic environments (Ulloa et al., 2012). It is likely that such *Alcanivorax* related OTU_97_s lack the capacity to outcompete strict aerobes in the oxic condition and adopt a fast growing strategy (r-strategist) when oxygen becomes available in the anoxic condition, which is a typical lifestyle in unstable and unpredictable environments (Andrews and Harris, 1986). These OTU_97_s were closely related to a sequence retrieved in desalination plant (IDA484: clone UV-RV-025, acc. no. HQ326427) and a sequence detected in deep-sea petroleum contaminated sediment (ID.A0, ID.A9793, ID.A8753: clone TVG01-83, acc. no. KF545057). The presence of different *Alcanivorax* related OTU_97_s is in accordance with previous reports showing the functional redundancy of hydrocarbon degradation within the *Alcanivorax* genus (Barbato et al., 2016). *Alcanivorax* species have been shown to exploit distinct hydrocarbon substrates as carbon and energy sources following different physiological strategies (Barbato et al., 2016) and presenting different susceptibilities to hydrostatic pressure (Scoma et al., 2016). Such metabolic diversity may explain niche differentiation as observed for Alcanivorax phylotypes, phylotype SK2 occupying floating biofilms while phylotype OM-2 living in the sediment (Coulon et al., 2012). In our study, the dissimilar behavior shown by OTU_97_s ID.A9793 and ID.A8783 between the two oxygenated phases suggested the presence of subpopulations. *Cycloclasticus*, which was also among the most abundant genus, was represented by OTU_97_s showing different behavior (**Figure 3B**). Eight OTU_97_s (ID.C9747, ID.C3, ID.C8389, ID.C4039, ID.C7228, ID.C1181, ID.C178, ID.C2805) were detected under all conditions, but they were most abundant under permanent oxic condition. These OTU_97_s were related to *Cycloclasticus pugetii* 15BN12L-10 (acc. no. KF470997) isolated from Artic Ocean deep-sea hydrocarbon-contaminated sediment (ID.C9747, ID.C3 and ID.C8389), sequences of clones (acc. nos. AM882527, EU438147 and KJ094255) detected in petroleum-contaminated coastal marine sediments (ID.C4039, ID.C178 and ID.C2805 respectively), sequences of clones (acc. nos. FJ981469 and FJ980914) obtained from deep-sea hydrothermal plume (ID.C7228 and ID.C1181 respectively). Two OTU_97_s (ID.C5592 and ID.C7005) were detected under both permanent anoxic and permanent oxic conditions while the OTU_97_s ID.C9052 was not detected under the permanent anoxic condition. These OTU_97_s were closely related to *Cycloclasticus pugetii* 15BN12L-10 (ID.C7005: acc. no. KF470997), extracellular symbiont BG-C1 of *Benthomodiolus* (ID.C5592: acc. no. AB679348) and a sequence obtained in petroleum-contaminated coastal sediment (ID.C9052: acc. no. FM242294). *Cycloclasticus* was more sensitive to anoxic/oxic oscillations than *Alcanivorax* (**Figure 3**). Although this condition was unfavorable for its optimal growth, *Cycloclasticus* showed almost constant low abundances with only slight increases during the oxygenated phases, behavior that is more typical of K-strategist with slow growth. Such a strategy may explain the metabolic activity of some PAH-degrading populations that were shown previously to express *rhd* gene transcripts even in absence of oxygen during anoxic/oxic oscillations (Vitte et al., 2013). Because *Cycloclasticus* species been described as obligate aerobes (Staley, 2010), the presence of a single OTU_97_ in all conditions may suggest the existence of distinct ecotypes, which correspond to a subpopulation that has acquired the genetic capacity to inhabit a slightly different ecological niche (Konstantinidis and Tiedje, 2005).

### *Alcanivorax* and *Cycloclasticus* Micro-Diversity

The micro-diversity of *Alcanivorax* and *Cycloclasticus* was examined in order to determine whether ecotypes explain their distribution in ecological niches distinguished by their oxygen availability. The resolution of 16S rRNA gene transcript sequences at the subpopulation level allows the identification of specific ecotypes inhabiting distinct ecological niches (Eren et al., 2013; Tikhonov et al., 2015). We acknowledge the inherent biases of 16S rRNA gene-based approaches to define ecotypes, particularly the limited capacity in defining phylogenetic cohesive populations (Berry et al., 2017) and functional traits (Martiny et al., 2013). But such 16S rRNA-based approaches provide useful information to draw hypotheses that could explain observed ecological behavior of hydrocarbon-degrading bacteria.

**FIGURE 4:**
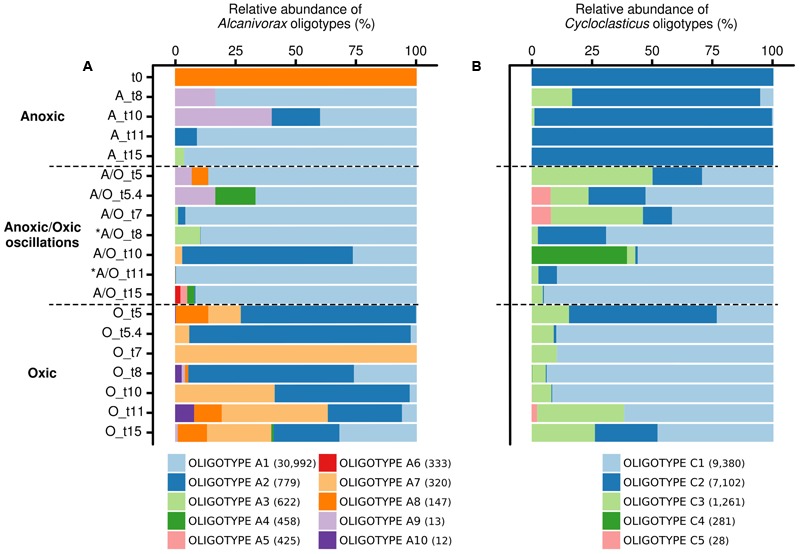
Relative abundances of *Alcanivorax* oligotypes **(A)** and *Cycloclasticus* oligotypes **(B)** according to oxygenation regimes. Oligotyping analysis was performed on non-normalized sequences. Oligotypes correspond to distinct subpopulations of ecological importance. The distribution of oligotypes was followed in permanent anoxic, anoxic/oxic oscillation and permanent oxic conditions. The number of sequence for each oligotype is indicated in brackets. The analysis is based on biological triplicates. ^∗^ indicates oxygenation period under anoxic/oxic oscillation condition.

**FIGURE 5:**
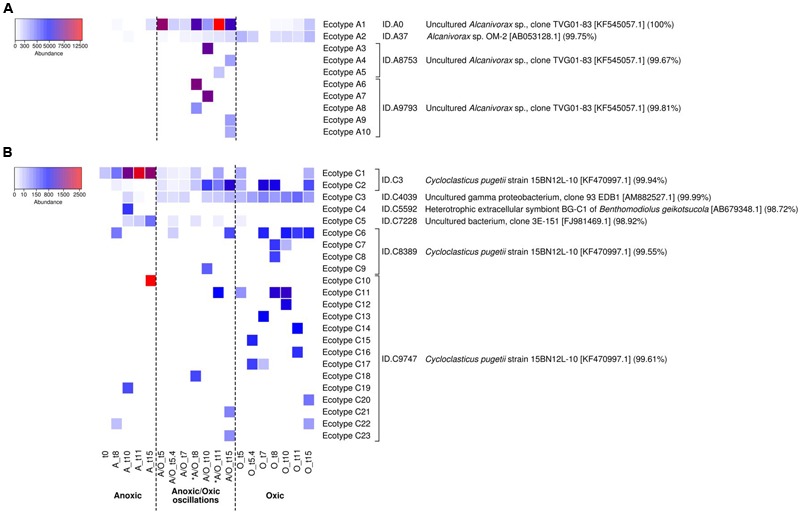
Relative abundances of *Alcanivorax* ecotypes **(A)** and *Cycloclasticus* ecotypes **(B)** according to oxygenation regimes. The heatmap was performed at the sub-species level (swarm dissimilarity threshold = 1 on *log*-transformed data) representing ecotypes, which correspond to specific cohesive subpopulations correlated to an oxygenation condition. Each ecotype represents more than 100 sequences. The color legend at the left indicates the ecotype abundance (sequence counts). ID sequences and their affiliation (highest BLAST hits) are indicated on the right. The analysis is based on biological triplicates. ^∗^ indicates oxygenation period under anoxic/oxic oscillation condition.

Oligotyping has proved useful for the detection of ecotypes in several occasions (Eren et al., 2013; Berry et al., 2017), particularly for hydrocarbon-degrading bacteria during the *Deepwater Horizon* oil spill (Kleindienst et al., 2016). Oligotyping of *Alcanivorax* and *Cycloclasticus* 16S rRNA gene transcript sequences pinpointed 10 and 5 oligotypes respectively. The distribution of *Alcanivorax* and *Cycloclasticus* oligotypes, which was consistent between biological replicates (PerMANOVA *p*-value > 0.05), showed different patterns according to the oxygenation regimes (**Figure 4**). The affiliation of oligotypes and their relation with OTU_97_s IDs and accession numbers of closest relatives species are presented in Supplementary Table 2. For *Alcanivorax*, oligotype A1 dominated under anoxic and anoxic/oxic oscillation conditions, while oligotype A2 dominated the oxic condition (**Figure 4A**). Oligotype A1 was related to the clone TVG01-83 (acc. no. KF545057 associated to OTU_97_s ID.A0, ID.A8753 and ID.A9793), closely related to the type strain *Alcanivorax borkumensis* SK2. Oligotype A2 was related to *Alcanivorax* sp. OM-2 (acc. no. AB053128 associated to OTU_97_ ID.A3). Additionally, *Alcanivorax* oligotypes were found specific for both anoxic (oligotypes A4, A5, A6 and A9, related to clone TVG01-83 associated to OTU_97_s ID.A0, ID.A8753 and ID.A9793) and oxic (oligotypes A7, A8 and A10, related to *Alcanivorax* sp. OM-2 associated to OTU_97_ ID.A3) samples. For *Cycloclasticus*, oligotypes C1 and C3 dominated under oxic and anoxic/oxic oscillation conditions whereas oligotype C2 was found dominant under anoxic condition (**Figure 4B**). These oligotypes were closely related to *Cycloclasticus pugetii* (acc. no. KF470997 associated to OTU_97_s ID.C3, ID.C9747 and ID.C8389) and sequence recovered from hydrothermal plume (acc. no. FJ981469 associated to OTU_97_ ID.C7228), extracellular symbiont BG-C1 of *Benthomodiolus* (acc. no. AB679348 associated to OTU_97_ ID.C5592) and hydrocarbon polluted coastal sediment (acc. no. AM882527 associated to OTU97 ID.C4039). Interestingly, oligotype C4 emerged specifically after the second oxygenation phase under the anoxic/oxic oscillation condition (**Figure 4B**), which was observed within all three biological replicates. This oligotype was affiliated to *Cycloclasticus pugetii* (acc. no. KF470997 associated to OTU_97_s ID.C3 and ID.C9747). The oligotypes explained only partly the behavior of *Alcanivorax* and *Cycloclasticus* related species to withstand oxygenation conditions. For example, it is not clear whether specialized ecotypes drive the success of *Alcanivorax* under anoxic/oxic oscillation since oligotype A1 was found dominant during all incubation period for both permanent anoxic and anoxic/oxic oscillation conditions. Similarly, although the distribution of *Cycloclasticus* oligotypes showed specialized ecotypes for anoxic permanent condition, the distribution of oligotypes C1 and C3 found under both anoxic and oxic samples suggested non-homogeneous subpopulations. We thus explored the micro-diversity of *Alcanivorax* and *Cycloclasticus* more deeply (swarm analysis with a dissimilarity threshold = 1), which revealed 10 and 23 subpopulations respectively (**Figure 5**). We assume that these subpopulations represent ecotypes since they occupy distinct ecological niches as reported by Tikhonov et al. (2015), who defined ecological subpopulations differing by one nucleotide in their 16S rRNA gene sequence. Furthermore, swarm analysis revealed cohesive subpopulations specifically observed under an oxygenation condition (**Figure 5**). PerMANOVA did not reveal difference in the distribution of *Alcanivorax* and *Cycloclasticus* ecotypes within biological replicates (*p*-value > 0.5). *Alcanivorax* ecotypes were related to the OTU_97_s ID.A9793 and ID.A8753 whereas the OTU_97_s ID.A0 and ID.A37 (ecotypes A1 and A2 respectively) showed uniform and cohesive populations irrespective of the oxygenation condition (**Figure 5A**). The ecotypes’ succession reflected the distinct ecological niches occurring throughout the incubation under the anoxic/oxic oscillation condition characterized not only by oxygen availability but also by metabolites appearing during aliphatic hydrocarbon degradation as well as the presence of other organic substrates. Ecotypes A6 (91.5% of oligotype A6) and A8 (100% of oligotype A3) related to OTU_97_ ID.A9793 emerged after the first oxygenation period while ecotype A5 (100% of oligotype A6) related to OTU_97_ ID.A8753 was observed after the second oxygenation period (**Figure 5A**). Ecotypes A3 (100% of oligotype A1) and A7 (94.6% of oligotype A1) bloomed during the second anoxic period while ecotypes A4 (100% of oligotype A5), A9 (100% of oligotype A4) and A10 (100% of oligotype A6) appeared at the end of the incubation after the third anoxic phase. Exploring the micro-diversity at one nucleotide difference level allowed the identification of specific ecotypes occupying distinct ecological niches during the incubation under anoxic/oxic oscillation condition. It is likely that such *Alcanivorax* ecotypes may take advantages of oxygen-fluctuating hydrocarbon-contaminated environments allowing them to compete with other alkane-degraders such as *Marinobacter* and *Thalassolituus* detected in our study at lower abundances (0.27 and 0.51% of total OTU_97_s, respectively), and probably with other alkane-degraders such as *Oleibacter, Oleispira* and *Colwellia* (all below 0.005% in our study), which have been detected in hydrocarbon-contaminated environments (Coulon et al., 2012) including during *Deepwater Horizon* oil spill (Kleindienst et al., 2016; Yang et al., 2016a).

For *Cycloclasticus*, the OTU_97_s ID.C4039, ID.C5592 and ID.C7228 (ecotypes C3, C4, and C5 respectively) showed cohesive populations (**Figure 5B**). *Cycloclasticus* ecotypes reflecting the succession of ecological niches during incubations under permanent oxic condition (ecotypes C12, C13, C14, C15, C16, C17, and C20 related to OTU_97_s ID.C9747 and ecotypes C7 and C8 related to OTU_97_s ID.C8389), permanent anoxic condition (ecotypes C10 and C19 related to OTU_97_s ID.C9747) and anoxic/oxic oscillation condition (ecotypes C11, C18, C21, and C23 related to OTU_97_s ID.C9747 and ecotype C9 related to OTU_97_s ID.C8389) were revealed (**Figure 5B**). Additional ecotypes present under both aerobic and anaerobic conditions related to OTU_97_s ID.C9747 (ecotype C22) and OTU_97_s ID.C8389 (ecotype C6) were also identified. All these ecotypes (except ecotype C22, 100% of sequences to oligotype C3) were linked with either oligotype C1 (>70% of sequences; ecotypes C12, C13, C15, C16, C17, C18, and C23), oligotype C2 (100% of sequences; ecotypes C6, C7, C8, C9, C14, C20, and C21) or both oligotypes C1 and C2 (around 50% of each sequences; ecotypes C10 and C19) indicating that the micro-diversity analysis at one nucleotide difference level allowed further discrimination for specific ecotypes reflecting the succession of ecological niches characterized by substrates and oxygen availabilities. Two ecotypes related to OTU_97_s ID.C3 (ecotype C1 corresponding to 97% of oligotype sequences C2 and ecotype C2 corresponding to 100% of oligotype C1 sequences) also illustrate ecological-niche specificity, showing contrasting behavior, especially regarding the anoxic period under anoxic/oxic oscillation condition. Previous studies reported on *Cycloclasticus* ecotypes detected specifically before, during and after *Deepwater Horizon* oil spill (Kleindienst et al., 2016; Yang et al., 2016b). Here we provide evidence of the coexistence of *Cycloclasticus* ecotypes linked to oxygenation conditions, which is useful information to further understand the presence of *Cycloclasticus* species in different environmental compartments, including the oil slick on the waters’ surface (Yang et al., 2016a), oiled marine snow (Arnosti et al., 2016), deep hydrocarbon plumes (Redmond and Valentine, 2012) and coastal sediments (Coulon et al., 2012; Stauffert et al., 2013; Yang et al., 2016b).

## Conclusion

An investigation of the behavior of *Alcanivorax* and *Cycloclasticus*, the most widely distributed MOHCB, under well-controlled different oxygenation regimes, revealed that members of these genera adopted distinct strategies to develop under oxygen-fluctuating conditions in oiled sediments. Anoxic/oxic oscillations were more favorable for *Alcanivorax*, which was more abundant than under the other conditions, suggesting that *Alcanivorax* behaved as a typical r-strategist. In contrast, *Cycloclasticus* abundance was lower in such fluctuating conditions compared with both permanent anoxic and permanent oxic conditions. Oligotyping revealed that the distribution of *Alcanivorax* and *Cycloclasticus* subpopulations (oligotypes) correlated with the redox conditions, but the analysis was unable to fully explain how these hydrocarbon-degraders may withstand anoxic/oxic oscillations. Further micro-diversity analysis at one nucleotide difference level (swarm analysis) allowed the identification of specific subpopulations, which we assume correspond to ecotypes occupying distinct ecological niches during the incubation under anoxic/oxic oscillation condition characterized by substrates and oxygen availabilities. Such ecotypes allow the colonization of distinct ecological niches that may explain the success of these MOHCB genera during an oil-spill. However, further efforts are required to isolate and characterize *Alcanivorax* and *Cycloclasticus* ecotypes to gain new insights on their ecological role within microbial networks involved in oil degradation in marine environments.

## Author Contributions

RD, CC-L, and CC conceived and designed the study. RD, CC-L, CC, and FT ran the experiments. RD, CC-L, CC, FT, CN, AD, and TMG analyzed the resulting data. RD wrote the manuscript. CC-L, CN, and CC revised the manuscript.

## Conflict of Interest Statement

The authors declare that the research was conducted in the absence of any commercial or financial relationships that could be construed as a potential conflict of interest.
