# Supplementary material for: Variation of Oxygenation Conditions on a Hydrocarbonoclastic Microbial Community Reveals Alcanivorax and Cycloclasticus Ecotypes
Source: Front Microbiol. 2017 Aug 16;8:1549. doi: 10.3389/fmicb.2017.01549 (PMC5562018; doi:10.3389/fmicb.2017.01549)
Supplement: Supplementary file 1 [file Data_Sheet_1.DOCX]

Supplementary Material

Variation of oxygenation conditions on a hydrocarbonoclastic microbial community reveals Alcanivorax and Cycloclasticus ecotypes

Fanny Terrisse^1^, Cristiana Cravo-Laureau^1^, Cyril Noel^1^, Christine Cagnon^1^, Alex Dumbrell^2^, Terry McGenity^2^, Robert Duran^1*^

^1^Equipe Environnement et Microbiologie, MELODY group, Université de Pau et des Pays de l’Adour, IPREM UMR CNRS 5254, BP 1155, 64013 Pau Cedex, France

^2^School of Biological Sciences, University of Essex, Wivenhoe Park Colchester CO43SQ, United Kingdom

*** Correspondence:** Corresponding Author: Robert.duran@univ-pau.fr

Figure 1: Oxygenation and redox status under the different conditions. Oxygen saturation (%; filled black squares connected by a solid black line) and redox potential (mV; filled grey squares connected by a dotted grey line) measured during incubations under permanent anoxic (A), anoxic/oxic oscillation (B) and permanent oxic (C) conditions. Error bars indicate the standard error (n=3).

Figure 2: Biodegradation indexes. nC17/Pristane (left) and Phenanthrene/Dimethylphenanthrene (right) for estimating hydrocarbons biodegradation capabilities during the incubations under permanent anoxic condition (filled squares connected by a short dotted black line), anoxic/oxic oscillation condition (filled diamonds connected by a solid black line) and permanent oxic condition (filled circles connected by a large dotted black line). The shaded zones correspond to the aerated periods under the anoxic/oxic oscillation condition. Stars show a significant difference according to Tukey HSD test (*p*-value < 0.05) compared to the value at 10 hours after oil addition (day 5) from the considered conditions. The lowercases show a significant difference (*p*-value < 0.05) between the different conditions with the same letter at the considered time. Error bars indicate the standard error (n=3).


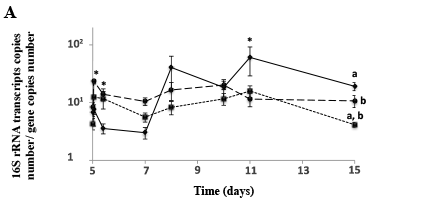

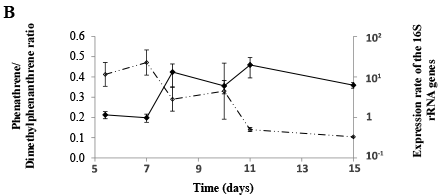

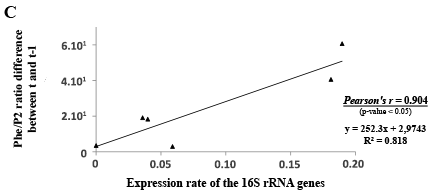


Figure 3: Correlation between 16S rRNA gene expression rate and phenanthrene biodegradation linked to oxygen addition. A) Expression rate of the 16S rRNA gene during incubation under permanent anoxic condition (filled squares connected by a short dotted black line), anoxic/oxic oscillation condition (filled diamonds connected by a solid black line) and permanent oxic condition (filled circles connected by a large dotted black line). Stars show significant difference according to Tukey HSD test (*p*-value < 0.05) compared to the value at 5 days, before oil addition from the considered conditions. The lowercases show a significant difference (*p*-value < 0.05) between the different conditions with the same letter at the considered time. B) Phenanthrene/Dimethylphenanthrene ratio (dotted black line) and 16S rRNA gene expression rate (solid black line) under anoxic/oxic oscillation condition. C) Pearson’s correlation straight line between diminution of the Phenanthrene/Dimethylphenanthrene ratio and the expression rate. The shaded areas correspond to the aeration periods under the anoxic/oxic oscillation condition. Error bars indicate the standard error (n=3).


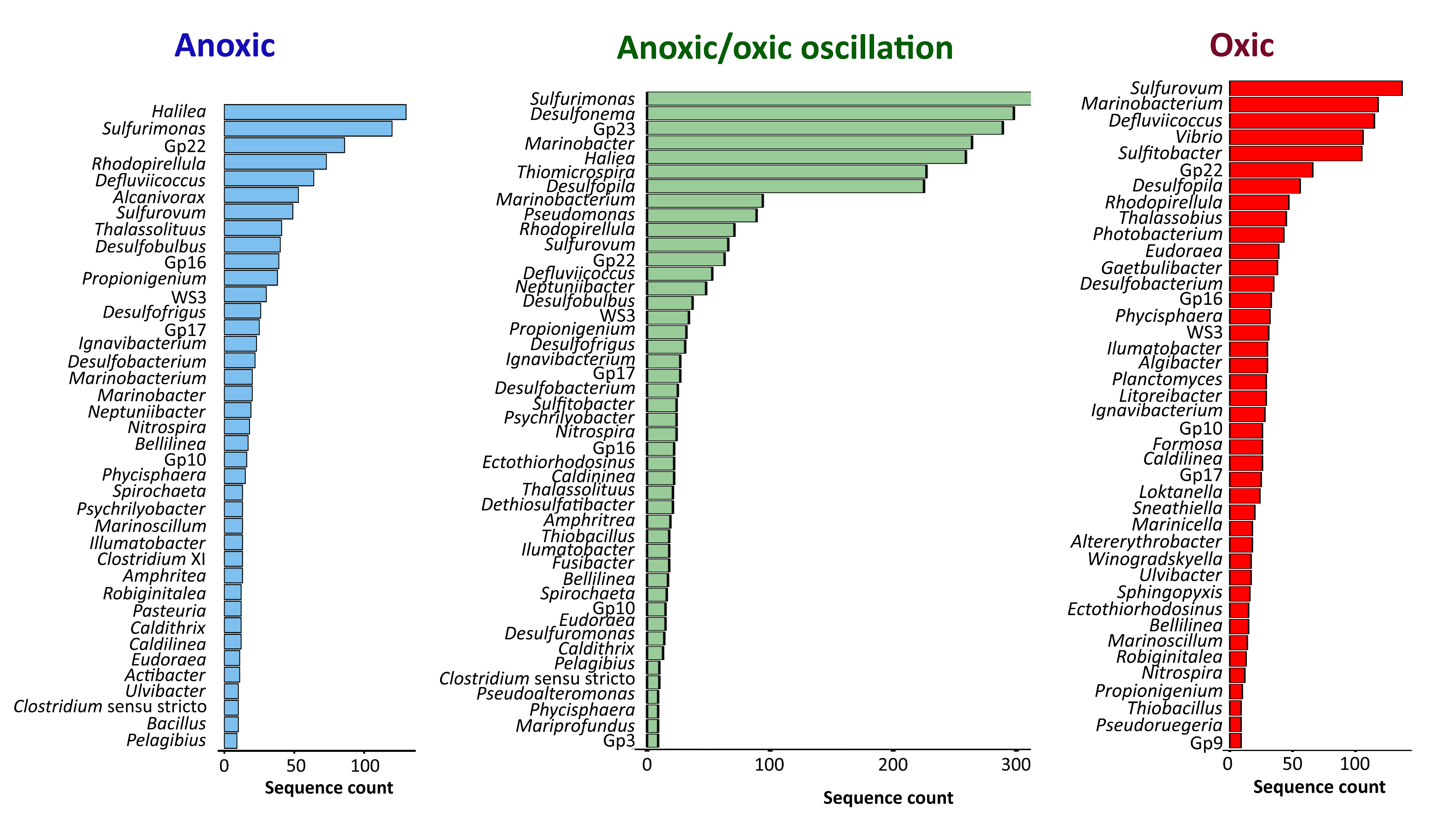


**Figure 4:** **Relative abundances of the OTU_97_s belonging to the "other group" defined in Fig. 3.** The relative abundances are presented for the permanent anoxic (right), the anoxic/oxic oscillation (middle) and the permanent oxic (left) conditions. The analysis was performed at the genus level applying a threshold similarity of 97% for OTU identification (OTU_97_s). The analysis is based on biological triplicates.

**Table 1: Diversity estimators of bacterial communities under the different oxygenation regimes: permanent anoxic, anoxic/oxic oscillation and permanent oxic conditions.** Richness and Shannon diversity indexes were determined applying a threshold similarity of 97 % (species level, OTU_97_) from bacterial (454-pyrosequencing) 16S rRNA gene transcript sequencing data. The analyses were performed in biological triplicates.

**Table 2: Relation between oligotypes, OTU_97_s IDs and accession numbers of closest relatives species for *Alcanivorax* (A) and *Cycloclasticus* (B).**

**A**

| **Oligotype** | **OTU_97_s** | **Closest relative** | **Accession no.** |
| --- | --- | --- | --- |
| A1 | ID.A0  ID.A8753  ID.A9793 | Uncultured *Alcanivorax* sp., clone TVG01-83 | KF545057.1 |
| A2 | ID.A3 | *Alcanivorax* sp. OM-2 | AB053128.1 |
| A3 | ID.A9793 | Uncultured *Alcanivorax* sp., clone TVG01-83 | KF545057.1 |
| A4 | ID.A0  ID.A8753  ID.A9793 | Uncultured *Alcanivorax* sp., clone TVG01-83 | KF545057.1 |
| A5 | ID.A0  ID.A8753  ID.A9793 | Uncultured *Alcanivorax* sp., clone TVG01-83 | KF545057.1 |
| A6 | ID.A0  ID.A8753  ID.A9793 | Uncultured *Alcanivorax* sp., clone TVG01-83 | KF545057.1 |
| A7 | ID.A3 | *Alcanivorax* sp. OM-2 | AB053128.1 |
| A8 | ID.A3 | *Alcanivorax* sp. OM-2 | AB053128.1 |
| A9 | ID.A0  ID.A8753  ID.A9793 | Uncultured *Alcanivorax* sp., clone TVG01-83 | KF545057.1 |
| A10 | ID.A3 | *Alcanivorax* sp. OM-2 | AB053128.1 |

**B**

| **Oligotype** | **OTU_97_s** | **Closest relative** | **Accession no.** |
| --- | --- | --- | --- |
| C1 | ID.C3  ID.C9747  ID.C8389  ID.C7228  ID.C5592 | *Cycloclasticus* *pugetii* strain 15BN12L-10  Uncultured bacterium, clone 3E-151  Extracellular symbiont BG-C1 of *Benthomodiolus* | KF470997.1  FJ981469.1  AB679348.1 |
| C2 | ID.C3  ID.C9747  ID.C8389  ID.C7228  ID.C5592 | *Cycloclasticus* *pugetii* strain 15BN12L-10  Uncultured bacterium, clone 3E-151  Extracellular symbiont BG-C1 of *Benthomodiolus* | KF470997.1  FJ981469.1  AB679348.1 |
| C3 | ID.C3  ID.C9747  ID.C8389  ID.C7228  ID.C5592 | *Cycloclasticus* *pugetii* strain 15BN12L-10  Uncultured bacterium, clone 3E-151  Extracellular symbiont BG-C1 of *Benthomodiolus* | KF470997.1  FJ981469.1  AB679348.1 |
| C4 | ID.C3  ID.C9747 | *Cycloclasticus* *pugetii* strain 15BN12L-10 | KF470997.1 |
| C5 | ID.C3  ID.C9747 | *Cycloclasticus* *pugetii* strain 15BN12L-10 | KF470997.1 |
